# Successful endoscopic closure and remove the clip from the peritoneal cavity for delayed perforation defect after duodenal endoscopic submucosal desection

**DOI:** 10.1055/a-2610-2697

**Published:** 2025-08-01

**Authors:** Qianqian Chen, Kunming Lyu, Jinping Li, Enqiang Linghu

**Affiliations:** 1 651943 Department of Gastroenterology, The First Medical Center of Chinese PLA General Hospital, Beijing, China; 2 670131 Department of Gastroenterology, The Second Medical Centre, National Clinical Research Centre for Geriatric Diseases, Chinese PLA General Hospital, Beijing, China

Duodenal endoscopic submucosal dissection (ESD) is still considered a high-risk procedure due to its complexity and potential complications. Compared with endoscopic submucosal dissection in other parts of the digestive tract, it has a significantly higher incidence of adverse events 1 . Traditional treatment methods for delayed perforation after duodenal ESD include duodenal stent placement, laparoscopic surgical repair, etc. 2 3 4 . However, when the complication of delayed perforation is accompanied by the detachment of clips into the abdominal cavity, these traditional treatment methods have disadvantages such as large trauma and a long recovery period. In a patient with duodenal delayed perforation, we not only removed the detached clip but also successfully repaired the defect endoscopically and achieved good clinical outcomes. This innovative endoscopic treatment method has the advantages of simple operation and minimal trauma and will provide a valuable supplementary solution for the treatment of digestive tract perforation combined with the detachment of foreign bodies.

A 71-year-old man was admitted to our hospital with a 0.5 × 1.5 cm submucosal tumor in the duodenal descending part and near the duodenal papilla. We resected the lesion using ESD, and the defect was sutured by clips. Two days later, the patient experienced the first delayed perforation after ESD. Endoscopic examination revealed clip detachment and perforation. Additional clips and a combined porcine fibrin sealant kit were given to seal the perforation. After conservative treatment with a Freka trelumina, the patient was discharged after 110 days.

Unfortunately, the patient experienced a second delayed perforation 133 days after ESD. Endoscopy examination revealed only two clips left at the duodenal mucosa closure site, with pus accumulated in the nearby bowel ( Fig. 1 **a** ). On closer inspection, a delayed duodenal perforation was found ( Fig. 1 **b** ). Computed tomography (CT) imaging revealed intra-abdominal fluid collections and demonstrated migration of a dislodged clip into the peritoneal cavity ( Fig. 1 **c** ). Given the complex peritoneal involvement and inherent surgical challenges associated with this complication, we attempted endoscopic repair ( Video 1 ). Under endoscopic observation, a catheter was placed in the perforated area and a contrast agent was administered via the tube. Under X-ray, the contrast agent was seen leaking through the perforation into the peritoneal cavity, and the position of the dislodged clip was clearly identified ( Fig. 1 **d** ). Through the duodenal perforation, we advanced a foreign body forceps into the peritoneal cavity, grasped the clip, and removed it ( Fig. 1 **e,f** ). Then, we used through-the-scope twin clips combined with clips to close the delayed perforation defect ( Fig. 1 **g** ). Freka trelumina was placed again with the suction port of the gastric tube keeping near the defect. A lot of peritoneal abscesses were formed after the second delayed perforation, and extracorporeal puncture drainage and regular flushing therapy were performed. Afterward, endoscopic observation of the perforation was repeated every 10 days until the defect was completely repaired ( Fig. 1 **h** ).

**Fig. 1:**
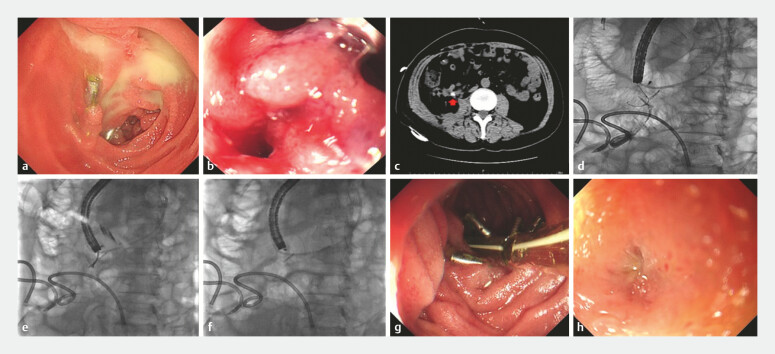
The endoscopic treatment process of duodenal perforation. **a** Endoscopy showed two clips left at the duodenal mucosa closure site, with pus accumulated in the nearby bowel. **b** Delayed duodenal perforation. **c** CT imaging revealed intra-abdominal fluid collections and demonstrated migration of a dislodged clip into the peritoneal cavity. **d** The position of the dislodged clip was clearly identified under X-ray. **e** The dislodged clip was grasped by the foreign body forceps. **f** The dislodged clip was removed. **g** The defect was completely closed by using through-the-scope twin clips in combination with clips. **h** Fully healed duodenal defect.

The endoscopic treatment process of duodenal perforation.Video 1

This case demonstrates the successful endoscopic management of delayed duodenal perforation and retroperitoneal abscess following duodenal ESD, resulting in complete recovery without surgical intervention. It offers a valuable reference for addressing the complication of delayed duodenal perforation.

Endoscopy_UCTN_Code_CPL_1AH_2AZ_3AD

## Notes

Endoscopy E-Videos https://eref.thieme.de/e-videos
*E-Videos* is an open access online section of the journal *Endoscopy* , reporting on interesting cases and new techniques in gastroenterological endoscopy. All papers include a high-quality video and are published with a Creative Commons CC-BY license. Endoscopy E-Videos qualify for HINARI discounts and waivers and eligibility is automatically checked during the submission process. We grant 100% waivers to articles whose corresponding authors are based in Group A countries and 50% waivers to those who are based in Group B countries as classified by Research4Life (see: https://www.research4life.org/access/eligibility/ ). This section has its own submission website at https://mc.manuscriptcentral.com/e-videos .
